# Complete Genome Sequences of *Lactobacillus curvatus* KG6, *L. curvatus* MRS6, and *Lactobacillus sakei* FAM18311, Isolated from Fermented Meat Products

**DOI:** 10.1128/genomeA.00915-17

**Published:** 2017-09-21

**Authors:** Christoph Jans, Sandra Lagler, Christophe Lacroix, Leo Meile, Marc J. A. Stevens

**Affiliations:** Laboratory of Food Biotechnology, Institute of Food, Nutrition and Health, ETH Zurich, Zurich, Switzerland

## Abstract

The genomes of *Lactobacillus curvatus* KG6, *L. curvatus* MRS6, and *Lactobacillus sakei* FAM18311 were sequenced and assembled using PacBio single-molecule real-time (SMRT) technology. The strains were isolated from Swiss fermented meat products. Circular chromosomes were of 1.98 Mbp (KG6), 2.11 Mbp (MRS6), and 1.95 Mbp (FAM18311), with a G+C content of 41.3 to 42.0%.

## GENOME ANNOUNCEMENT

*Lactobacillus curvatus* and *Lactobacillus sakei* are closely related species (1) associated with the human intestine and fermented meat products as important contributors to the production of salami-type sausages (2–4). *L. curvatus* KG6 was isolated on de Man-Rogosa-Sharpe (MRS) agar (Biolife, Milan, Italy) from a salami-type fermented meat product purchased at the retail level in Switzerland in 1999, and it showed phenotypic novobiocin resistance (5). *L. curvatus* MRS6 is a tyramine producer that was isolated at 10^6^ CFU ml^−1^ on MRS agar (Biolife) from the traditional Swiss fermented sausage “salsiz” made of deer meat purchased at the retail level in Switzerland in 2016 (J. Dürig, unpublished data). *L. sakei* FAM18311 is part of the FAM strain collection of Agroscope Liebefeld-Posieux (Liebefeld, Switzerland) originating from a fermented meat product (6). With the aim to establish *L. curvatus* and *L. sakei* strains for metabolic studies and the application of novel genetic tools, these strains were subjected to whole-genome sequencing.

An overnight culture was used to propagate the strains under anaerobic conditions at 37°C in MRS broth (Biolife) for DNA isolation. The Wizard genomic DNA purification kit (Promega, Madison, WI, USA) was used for DNA isolation, including modifications as previously described (7). One single-molecule real-time (SMRT) cell per strain was used for sequencing of the genomic DNA on a PacBio RSII system (Pacific Biosciences, Menlo Park, CA, USA) at the Functional Genomics Center Zurich (Zurich, Switzerland). Genome assembly of KG6 and MRS6 was performed using the SMRT Analysis system SMRT Portal (version 2.3.0.140936.p1.142411; Pacific Biosciences). The genome of FAM18311 was assembled using CLC Genome Finishing Module version 1.6.2 (Qiagen, Aarhus, Denmark). All genomes reached 300× coverage, assembled from 77,000 to 105,000 reads with an *N*_50_ read length between 23,000 and 32,000 nucleotides (nt). The genomes were annotated using the NCBI Prokaryotic Genome Annotation Pipeline. Ori-Finder 2 was used to predict the chromosomal origin of replication (8). Species designations were confirmed by average nucleotide identity calculations (9).

FAM18311 harbored a circular chromosome of 1,945,884 bp in length. FAM18311 harbored two plasmids, pFAM18311_1 and pFAM18311_2, of 84,581 bp and 26,098 bp, respectively. The chromosome of FAM18311 featured 1,896 predicted coding sequences (CDSs) and a G+C content of 41.3%. Plasmids pFAM18311_1 and pFAM18311_2 harbored 90 CDSs and 29 CDSs, with G+C contents of 34.5% and 40.4%, respectively. KG6 harbored a circular chromosome of 1,985,155 bp and a plasmid pKG6_1 of 17,609 bp, with G+C contents of 42.0% and 44.9%, respectively. The chromosome of KG6 and its plasmid pKG6_1 featured 1,970 and 17 predicted CDSs, respectively. MRS6 harbored a circular chromosome of 2,114,309 bp, with a G+C content of 41.7% and 1,975 predicted CDSs. MRS6 was previously found to produce tyramine (J. Dürig, unpublished data). The presumptive operon encoding tyramine production was located around the tyrosine decarboxylase-encoding gene (CG419_06595). *L. curvatus* MRS6 will thus provide a future metabolic model strain for genetic engineering of tyramine production pathways.

### Accession number(s).

This whole-genome project has been deposited at DDBJ/ENA/GenBank under the accession numbers CP020459 to CP020461 (FAM18311), CP022474 (MRS6), and CP022475 and CP022476 (KG6). The versions described in this paper are the first versions, CP020459.1 to CP020461.1 and CP022474.1 to CP022476.1.
